# Educational Attainment Better Reduces Disability for Non-Hispanic than Hispanic Americans

**DOI:** 10.3390/ejihpe10010002

**Published:** 2019-07-17

**Authors:** Shervin Assari, Mohsen Bazargan

**Affiliations:** 1 Department of Family Medicine, Charles R Drew University of Medicine and Science, Los Angeles, CA 90059, USA; 2 Department of Family Medicine, University of California Los Angeles (UCLA), Los Angeles, CA 90095, USA

**Keywords:** educational attainment, income, race, ethnicity, socioeconomic status, chronic disease, disability, higher education

## Abstract

Objectives: Minorities’ Diminished Returns (MDRs) refers to the weaker protective health effects of socioeconomic status (SES) for minorities, particularly educational attainment for racial and ethnic minorities, compared to the general population. This pattern has been documented among African-Americans compared to Whites, however, we know very little about MDRs for educational attainment on disability among Hispanics compared to Non-Hispanic Whites. Aims: This cross-sectional study explored ethnic variation in the effects of educational attainment on severity of disability in the United States of America (USA). Materials and Methods: The 2015 National Health Interview Survey (NHIS) was a national survey of the general population in the USA. The total sample was 1021 American adults that reported some disability. Of the 1021 participants, 855 identified as Non-Hispanic and 165 identified as Hispanic. The independent variable was educational attainment. The main outcome was severity of disability measured using self-reported data. Age, gender, and race were covariates. Ethnicity was the effect modifier. Results: Among individuals with a disability, higher levels of educational attainment were associated with fewer disabilities, independent of all confounders. When ethnicity and educational attainment were interacted on severity of disability, the results indicated a smaller protective effect for Hispanics than for Non-Hispanics with a disability. Ethnicity-stratified models showed an effect for Non-Hispanics but not for Hispanics. Conclusions: The protective effects of educational attainment against severity of disability are smaller for Hispanics than for Non-Hispanics. To prevent health disparities, there is a need to minimize MDRs of SES for ethnic minorities. To do so, there is a need for innovative economic, public, and social policies that are not limited to equalizing educational attainment but that also help minorities leverage their resources and gain tangible outcomes.

## 1. Background

Socioeconomic status (SES) indicators (e.g., educational attainment) are protective against undesired health outcomes [1,2,3]. Individuals with a higher educational attainment are at a lower risk of disability and morbidity defined in terms of chronic medical conditions (CMCs) [1,4,5,6,7,8,9,10]. As SES is a main determinant of morbidity [4,5,7,10,11], SES inequalities exist in prevalence of morbidity and disability [12].

The health effects of an SES indicator, such as educational attainment, differ widely among various locations and populations [13,14,15,16]. The effects of SES on morbidity, CMCs, and disability are also shown to vary between sub-populations [14,15]. According to the Minorities’ Diminished Returns (MDRs) [17,18], however, the health of members of marginalized groups [19] is not heavily influenced by SES indicators, particularly educational attainment. Most studies have compared African-American and White individuals, with less research having been conducted on differential health effects of educational attainment for other ethnicities [11,19,20]. For example, while income is shown to have a stronger effect on reducing morbidity for Whites than for Blacks [11], it is unclear if the same effect applies to the comparison of Hispanics and Non-Hispanics. 

To date, very little is known about MDRs among Hispanics [21,22]. In one study, occupation was associated with a larger effect on smoking for non-Hispanics than for Hispanics [23]. In a different study, educational attainment was associated with greater reduced frequency of binge drinking for Non-Hispanics than for Hispanics [21]. In another study, education, income, employment, and marital status of family members enhanced oral health for Non-Hispanics but not for Hispanics [22]. We are not aware of any studies comparing Hispanic and Non-Hispanic communities for the effects of educational attainment on disability. These MDRs are well established for African-Americans [11,19,20].

## 2. Aim

This cross-sectional study used the National Health Interview Survey (NHIS) data to study the ethnic heterogeneities in the effects of educational attainment on the severity of disability among American adults.

## 3. Methods

The 2015 National Health Interview Survey (NHIS) was used for this study. The NHIS is one of the primary sources of information on the health of American adults. The sample was limited to non-institutionalized US civilians. Data collection was done by the National Center for Health Statistics (NCHS), Centers for Disease Control and Prevention (CDC).

### 3.1. Sampling

The NHIS data collection has been conducted in a continuous manner throughout the years. The NHIS sampling and sample design is available at the study website at https://www.cdc.gov/nchs/nhis/index.htm. NHIS uses a multi-stage sampling process that requires adjustment for participants’ weights. The first stage of the current multi-stage sample design involves sampling 428 of 1900 geographically defined primary sampling units (PSUs), with PSUs from all 50 USA states and the District of Columbia. A PSU may be either a small group of contiguous counties, a single county, or a metropolitan statistical area.

The NHIS has four main Cores that consist of: (1) Household Composition, (2) Family Core, (3) Sample Child Core, and (4) Sample Adult Core.

The US Census Bureau collects the NHIS data under a contractual agreement. Interviews on the NHIS are face-to face and occur in the participants’ households. These interviews are sometimes followed by, and on rare occasions replaced by, a telephone interview.

### 3.2. Participants

The sample in this study consisted of 1021 American adults that reported some disability. Of the 1021 participants, 855 identified as Non-Hispanic and 165 identified as Hispanic.

### 3.3. Measures

#### 3.3.1. Predictor

*Educational Attainment (EA).* Education was operationalized as a continuous variable with a range between 0 and 21, indicating the number of years of schooling participants had received. A higher score reflects higher educational attainment.

#### 3.3.2. Moderator

*Ethnicity.* Ethnicity was self-identified. All of the participants were either Non-Hispanic White or Non-Hispanic African-American, or Hispanic. Ethnicity was treated as a dichotomous variable (Hispanics = 1, Non-Hispanics = 0 (reference category)).

#### 3.3.3. Covariates

Demographic characteristics in the current study included race, age, gender, income, and marital status.

*Age.* Age was a continuous measure.

*Gender.* Gender was a dichotomous variable (male = 1, female = 0 (reference group)).

*Race.* All participants self-identified their race. Race was operationalized as a categorical variable (Blacks/African Americans = 1, Whites = 0 (reference category)).

#### 3.3.4. Dependent Variable

*Severity of Disability.* Severity of disability was self-reported and was measured using the following eight items: (a) Does - - need help with personal care?; (b) Does - - need help with bathing/showering?; (c) Does - - need help dressing?; (d) Does - - need help eating?; (e) Does - - need help in/out of bed or chairs?, (f) Does - - need help using the toilet?; (g) Does - - need help to get around in the home?; and (h) Does - - need help with routine needs? A sum score was calculated with a potential range from 0 to 8, with a higher score indicative of more severe disability.

### 3.4. Statistical Analysis

To accommodate the NHIS’s multi-stage sampling design, we applied SPSS 23.0 (IBM Inc., NY, USA) to perform data analysis. This enabled us to adjust for the NHIS survey weights that come from the design variable (strata, clusters, and non-response). Taylor series linearization was applied for re-estimation of the standard errors (SEs). For descriptive statistics, we used weighted means and frequencies. For multivariate analysis, we used linear regression models.

In our linear regression models, we used educational attainment as the independent variable, disability as the dependent variable, socio-demographics as control variables, and ethnicity as the focal moderator. The first two linear regression models were estimated in the pooled sample that included both Hispanics and Non-Hispanics. *Model 1* did not include ethnicity by the educational attainment interaction term. *Model 2* included ethnicity by the educational attainment interaction term. Subsequently, we performed ethnic-specific linear regressions (*Model 3* for non-Hispanics and *Model 4* for Hispanics). Adjusted *b*-values, 95% Confidence Intervals (CI), and *p*-values were reported. A *p*-value of less than 0.05 was considered significant.

### 3.5. Justification for Performing Four Models

Past studies on MDRs have used the same modeling strategy. However, the results of Model 2 may raise some questions. We needed four models for the following reasons. First, to test interactive models, we should always test the additive model first. As a result, we tested Model 1 first. Second, although Model 3 and Model 4 show the association of interest across groups, they do not provide any evidence for a “statistically significant difference” between the effect of our predictor on our outcome between the two groups. Third, our inference regarding statistically significant differences in slopes was based on the model in the pooled sample (Model 2), which was not affected by low statistical power. The interaction between group membership and our predictor suggests differences across groups that are not due to chance and are significant at 0.05 level. However, readers should not be misled by the sizes of the main effects (ethnicity and educational attainment), since they should not be interpreted independently and individually interpreted in this model. The only conclusion from Model 2 is that the effect of educational attainment on disability are significantly different across ethnic groups, with Hispanics showing a smaller effect compared to Non-Hispanics.

## 4. Results

### 4.1. Descriptive Statistics

The total sample in this study was 1021 American adults with disability. From this number, 855 were Non- Hispanic and 165 were Hispanic. Table 1 provides a summary of the descriptive statistics of the participants overall, and by ethnicity. Hispanics were considerably younger than non-Hispanics. As a result, Hispanics had fewer disabilities than non-Hispanics. Hispanics also had lower educational attainment than non-Hispanics.

### 4.2. Pooled Sample Linear Regressions

Table 2 shows the results of two linear regression models that were conducted in the pooled sample, with educational attainment as the independent variable and disability as the dependent variable. *Model 1* only included the main effect of our independent variables and covariates. *Model 2* also included an interaction term between ethnicity and educational attainment. Based on the results from *Model 1*, high educational attainment had protective effects against disability above and beyond our covariates. *Model 2* showed a significant interaction between ethnicity and educational attainment on disability, suggesting that the protective effect of educational attainment against disability is smaller for Hispanics than for non-Hispanics (Table 2).

### 4.3. Ethnic-Specific Linear Regressions

Table 3 summarizes the two linear regression models that were estimated for Non-Hispanics and Hispanics. *Model 3* and *Model 4* showed that with both Non-Hispanics and Hispanics, high educational attainment was associated with less disability; however, the magnitude of this association was stronger for Non-Hispanics than for Hispanics (Table 3).

## 5. Discussion

The current study showed that higher educational attainment was associated with fewer disabilities for the pooled sample. However, this effect was only significant for Non-Hispanics and not for Hispanics. That is, as educational attainment increases, people with disability report less severe disability, although this holds only for Non-Hispanics. This is a representation of minorities’ diminished returns (MDRs) of educational attainment on disability among Hispanics, relative to Non-Hispanics, in the presence of disability.

This finding is in line with other studies showing that SES indicators generate less than expected outcomes for Blacks, Hispanics, and sexual minorities. In fact, minority status, regardless of its type, whether based on race [1], ethnicity [21,22], or sexual orientation [24], is consistently associated with diminished health returns from the same resources [25].

These consistent patterns suggest that society treats marginalized groups differentially, and it is this differential treatment of marginalized groups that reduces their full participation in society. It is the social structure that does not allow minority groups to fully leverage their resources and gain the highest level of tangible outcomes from the same social resources [17,18].

Previous research has shown that education [26], income [11], occupation [27], and marital status [28] generate less health benefits for Blacks than Whites. The effects of education and income on CMCs [11] such as asthma [29], obesity [19,30] and ADHD [20] are smaller for Blacks than for Whites. None of these MDR patterns were previously documented for Hispanics compared to Non-Hispanics. The contribution of this study is to expand the literature on MDRs to Hispanics.

The observed ethnic differences in the protective effect of educational attainment against severity of disability in individuals with some disability may be due to inequalities across US institutions, including but not limited to the education system and the labor market. Scarcity of educational resources in predominantly Hispanic areas may be one reason we observe differential effects of education for ethnic groups. The educational system also discriminates against Hispanic children and youth, which reduces their chances of flourishing in school and preventing them from gaining the most from the education system. The ability of education to positively impact health depends on how easily education can be translated into employment, income and wealth, and that in turn depends on ethnicity. Despite anti-discrimination regulations, the labor market is still discriminating against non-Whites and offers worse employment opportunities for ethnic groups in comparison with Non-Hispanic Whites. In addition, due to residential segregation, Hispanics may face extra difficulty commuting to work and competing for low-stress high-paying jobs. Non-Hispanic Whites are more likely to obtain jobs with higher wages, good working conditions, and more opportunities for advancement. Hispanics and Blacks are more likely to obtain jobs with poor working conditions, high stress, and fewer opportunities for growth and promotion. As a result, even highly educated Hispanics may not enjoy equal access to the opportunity structure compared to their Non-Hispanic White counterparts [26,31]. All these mechanisms reduce the health gains of educational attainment for Non-Whites.

## 6. Limitations

Our study has some methodological limitations. A cross-sectional design prevents us from drawing causal inferences between educational attainment and disability. Reverse causality between disability and educational attainment cannot be ruled out. As with other studies comparing ethnic groups for the associations between the same risk factors and disability, this study may be affected by some biases. We cannot rule out differential validity of our disability measure across ethnic groups. Some may argue that conventional disability measures have a higher validity among Non-Hispanics than among Hispanics. In addition, we did not assess each type of disability, classes of disability, or their links to CMCs.

The results of the current analysis should be interpreted with caution also because: (1) the data were limited to a single year of the NHIS (2015); (2) we only compared Non-Hispanics and Hispanics; and (3) the number of control variables was limited. We only used 2015 data because we were more familiar with the structure of the survey and survey variables in that year. Other researchers may test similar hypotheses over a span of several years. In addition, future research should include other minority groups which, according to MDRs, should follow the pattern seen among Hispanics. As the existing literature on Blacks is very large, we did not feel the need to test the hypothesis for Blacks. In a review study published in 2018, about 40 papers had shown MDRs for Blacks. Other race and ethnic groups such as Native Americans, subgroups of Hispanics, and Asian Americans are still understudied and require further attention. Finally, this study did not measure a wide range of potential confounders such as access to the health care system. For instance, this study did not include area level measures such as density of resources and neighborhood measures.

Given these limitations, the conclusions that can be drawn from these analyses are limited. The findings are preliminary and only suggestive. Further research is needed to replicate and validate these findings in other settings, cohorts, and age groups.

## 7. Conclusions

The protective effects of educational attainment on the severity of disability is smaller for Hispanics with disabilities than for Non-Hispanics with disabilities. To prevent health disparities, it is imperative to minimize minorities’ diminished returns of educational attainment. To achieve this goal, we must implement innovative economic, public, and social policies that are not limited to equalizing SES, but that are aimed at helping minorities leverage their resources so as to gain tangible outcomes from them.

## 8. Ethics

All participants provided written consent and the ethics of the NHIS protocol was approved by the CDC IRB. According to the NIH guideline and the decision tool regarding human subject involvement of secondary analyses of existing data, the current study was found to be “Not Human Subject Research”. The definition and also the decision tool which was applied is also available here: https://grants.nih.gov/policy/humansubjects/hs-decision.htm. According to the of human research involvement in research, this study is found to be exempt from the IRB approval process by the CDU IRB.

## Figures and Tables

**Table 1:** Descriptive statistics of the participants with disability overall, and by ethnicity.

	All		Non-Hispanics		Hispanics	
	n	%	N	%	n	%
**Gender** *						
Female	646	63.3	526	61.5	120	72.7
Male	374	36.7	329	38.5	45	27.3
**Race** *						
Others	700	79.5	604	77.2	96	98.0
**Black**	180	20.5	178	22.8	2	2.0
Age (Years) *	66.36	16.93	66.78	16.94	64.18	16.73
Education (Years) *	13.09	4.32	13.67	3.85	10.09	5.33
Disability (n) *	4.36	2.03	4.29	2.01	4.70	2.12

* *p* < 0.05 for comparison of Hispanics and Non-Hispanics.

**Table 2:** Linear regressions between educational attainment and severity of disability in the pooled sample of adults with disability.

	B	95% CI		*p*
**Model 1**				
Ethnicity (Hispanic)	0.18	−0.27	0.63	0.428
Race (Black)	0.26	−0.09	0.60	0.142
Gender (Male)	−0.06	−0.34	0.22	0.666
Age (Years)	0.00	−0.01	0.01	0.375
Educational Attainment (Years)	−0.07	−0.10	−0.03	0.000
Married	−0.03	−0.10	0.03	0.336
Constant	5.00	4.10	5.90	0.000
**Model 2**				
Ethnicity (Hispanic)	−1.18	−2.28	−0.09	0.035
Race (Black)	0.24	−0.11	0.58	0.175
Gender (Male)	−0.06	−0.34	0.22	0.668
Age (Years)	0.00	−0.00	0.01	0.286
Educational Attainment (Years)	−0.09	−0.13	−0.049	0.000
Married	−0.03	−0.10	0.03	0.320
Ethnicity * Educational Attainment (Years)	0.12	0.03	0.21	0.008
Constant	5.29	4.37	6.21	0.000

**Table 3:** Summary of linear regressions between educational attainment and severity of disability in Hispanic and Non-Hispanic adults with disability.

	b	95% CI		*p*
**Model 3**				
Race (Black)	0.200	−0.145	0.545	0.256
Gender (Male)	−0.042	−0.334	0.251	0.779
Age (Years)	0.003	−0.006	0.012	0.474
Educational Attainment (Years)	−0.090	−0.129	−0.051	0.000
Marital Status	−0.041	−0.112	0.030	0.260
Constant	5.424	4.475	6.373	0.000
**Model 4**				
Race (Black)	3.193	0.200	6.185	0.037
Gender (Male)	−0.308	−1.300	0.684	0.539
Age (Years)	0.019	−0.008	0.047	0.168
Educational Attainment (Years)	0.051	−0.037	0.139	0.249
Marital Status	0.038	−0.169	0.245	0.714
Constant	2.611	−0.002	5.223	0.050
